# Ferroptosis as a Novel Determinant of *β*-Cell Death in Diabetic Conditions

**DOI:** 10.1155/2022/3873420

**Published:** 2022-03-14

**Authors:** Ana Stancic, Tamara Saksida, Milica Markelic, Milica Vucetic, Ilijana Grigorov, Vesna Martinovic, Dragica Gajic, Andjelija Ivanovic, Ksenija Velickovic, Nevena Savic, Vesna Otasevic

**Affiliations:** ^1^Department of Molecular Biology, Institute for Biological Research “Siniša Stanković”, National Institute of Republic of Serbia, University of Belgrade, Belgrade, Serbia; ^2^Department of Immunology, Institute for Biological Research “Siniša Stanković”, National Institute of Republic of Serbia, University of Belgrade, Belgrade, Belgrade, Serbia; ^3^Department of Cell and Tissue Biology, Faculty of Biology, University of Belgrade, Belgrade, Serbia; ^4^Medical Biology Department, Centre Scientifique de Monaco (CSM), Monaco

## Abstract

The main pathological hallmark of diabetes is the loss of functional *β*-cells. Among several types of *β*-cell death in diabetes, the involvement of ferroptosis remains elusive. Therefore, we investigated the potential of diabetes-mimicking factors: high glucose (HG), proinflammatory cytokines, hydrogen peroxide (H_2_O_2_), or diabetogenic agent streptozotocin (STZ) to induce ferroptosis of *β*-cells *in vitro*. Furthermore, we tested the contribution of ferroptosis to injury of pancreatic islets in an STZ-induced *in vivo* diabetic model. All *in vitro* treatments increased loss of Rin-5F cells along with the accumulation of reactive oxygen species, lipid peroxides and iron, inactivation of NF-E2-related factor 2 (Nrf2), and decrease in glutathione peroxidase 4 expression and mitochondrial membrane potential (MMP). Ferrostatin 1 (Fer-1), ferroptosis inhibitor, diminished the above-stated effects and rescued cells from death in case of HG, STZ, and H_2_O_2_ treatments, while failed to increase MMP and to attenuate cell death after the cytokines' treatment. Moreover, Fer-1 protected pancreatic islets from STZ-induced injury in diabetic *in vivo* model, since it decreased infiltration of macrophages and accumulation of lipid peroxides and increased the population of insulin-positive cells. Such results revealed differences between diabetogenic stimuli in determining the destiny of *β*-cells, emerging HG, H_2_O_2_, and STZ, but not cytokines, as contributing factors to ferroptosis and shed new light on an antidiabetic strategy based on Nrf2 activation. Thus, targeting ferroptosis in diabetes might be a promising new approach for preservation of the *β*-cell population. Our results obtained from *in vivo* study strongly justify this approach.

## 1. Introduction

Diabetes mellitus is a complex metabolic disorder with a rising incidence, which suggests the urgent need for finding promising therapeutic targets and approaches. The main pathological hallmark of diabetes (both type 1 and type 2) is the decrease of *β*-cell mass. So, there is a great effort nowadays to find a way to restore normal *β*-cell mass under diabetic conditions either by stimulating their proliferation, differentiation/transdifferentiation, or by inhibiting the cell death process. In searching for a successful approach to prevent/diminish diabetes progression, it has become of utmost significance to reveal molecular targets and signaling pathways underlying *β*-cell loss. Until now, several important regulators of *β*-cell destiny, responsible for their survival and death, were described. Among others, pancreatic and duodenal homeobox 1 (PDX-1) and mammalian sterile 20-like kinase 1 (MST1) play a central role in *β*-cell survival [1] and apoptosis [2]. Deficiency of the factors controlling PDX-1 and MST1, such as catechol-o-methyltransferase and its estrogen metabolite 2-methoxyestradiol [3], could underlie *β*-cell dysfunction and be a target of some antidiabetic drugs, such as metformin [4].

In addition, targeting *β*-cell death has also emerged as an important potential approach in diabetes treatment. Till date, several types of death of *β*-cells have been described in diabetic conditions including necrosis, apoptosis, and autophagy [5–8]. The connecting link between them is the disturbance of the redox state, suggesting that manipulation of the reactive species production/removal can have great therapeutic potential for the regulation of *β*-cell mass. Our previous results and results from others unequivocally showed that reactive species of both oxygen and nitrogen (ROS and RNS, respectively) affect the number of functional *β*-cells [5–7]. The most likely reason why *β*-cells are extremely sensitive to oxidative insults is an intrinsically low expression and activity of antioxidant enzymes compared to other cells [9]. When compared to the liver, pancreatic islets have significantly lower catalase, glutathione peroxidase (GPX), and superoxide dismutase 1 (SOD1) expression levels and activities [10–12]. What makes the situation even worse in the diabetic state is the suppression of antioxidant capacity along with the increase in the production of ROS in many tissues, including the pancreas [8, 13, 14].

Although up to date the main role in the impairment of pancreatic islet number has been ascribed to apoptosis, recent discoveries might challenge this point of view. Namely, in 2012, the group from the Columbia University contextualized a new type of cell death and coined the term “ferroptosis” for it [15]. Ferroptosis is a regulated, iron-dependent type of cell death whose backbone makes glutathione- (GSH-) GPX4-membrane hydroperoxide axis. Under the homeostatic conditions, oxidative damages occurring at the level of membrane lipids (lipid peroxides) are promptly removed by the GPX4 enzyme using GSH as a reducing power. However, if this antioxidant pathway becomes disturbed, membrane lipid peroxidation propagates throughout the membrane, compromising its integrity and finally resulting in cell death. So far, it has been shown that ferroptosis is involved in the pathogenesis of several diseases, including neurodegenerative diseases, cardiovascular diseases, and ischemia/reperfusion injuries in the brain, heart, and kidney [16–19], and even progress has been made in treating some of these diseases with ferroptosis modulators [20, 21]. However, the role of ferroptosis in diabetes (ethio)pathology is still an unexplored issue that has been suggested recently [8]. Few independent studies provided evidence for ferroptotic phenotype in the kidney [22, 23], bone [24], and pancreas [25, 26] in the *in vivo* models of diabetes. There, high glucose (HG) has been recognized as a causative factor of ferroptosis as it has been shown in proximal tubular epithelial cells, osteoclasts, and *β*-cells *in vitro* [22, 24–26]. However, it is still unknown how other pathological parameters related to diabetes interfere with this type of cell death and what are the main signaling pathways associated with it.

It is well known that diabetes is a complex and multifactorial pathology and that pathological milieu in the pancreas/*β*-cells during the progression of the disease involves metabolic and inflammatory components. Thus, hyperglycemia and hyperlipidemia, on the one hand, and proinflammatory cytokines on the other, play an important role in the regulation of *β*-cell destiny in the diabetic state. The effects of all mentioned pathological parameters correlate with increased production of ROS in *β*-cells either through enzymatic (nicotinamide adenine dinucleotide phosphate (NADPH) oxidase-dependent) or nonenzymatic pathways [5, 27, 28]. According to this, the diabetogenic capacity of various pharmacological agents commonly used in diabetes induction, such as streptozotocin (STZ), is based on the increase in oxidative stress [29] and ascribes to apoptosis of *β*-cells [14].

To shed more light on the role of ferroptosis in the regulation of *β*-cell loss in diabetes, we aimed to examine here whether the death of Rin-5F pancreatic cells induced by various diabetes-mimicking factors (high glucose (HG), proinflammatory cytokines, and hydrogen peroxide (H_2_O_2_)) and by the pharmacological agent for induction of diabetes (STZ) is ferroptotic by its nature. Furthermore, we aimed to test our hypothesis in STZ-induced *in vivo* diabetic model.

## 2. Material and Methods

### 2.1. Chemicals

RPMI 1640 medium with L-glutamine (RPMI-HA) and fetal bovine serum (FBS) (FBS-16A) were purchased from Capricorn Scientific (Germany). Penicillin and streptomycin (L0022-100) were obtained from Biowest (MO, USA). Ras-selective lethal small molecule 3 (RSL-3) (SML2234), ferrostatin-1 (Fer-1) (SML 0583), STZ (S0130), propidium iodide (PI) (P4864), H_2_O_2_ (H1009), trypsin (T3924), Tween 20 (P9416), sodium dodecyl sulfate (SDS) (L3771), dithiothreitol (DDT) (43815), and protease inhibitor cocktail (P8340) were purchased from Sigma-Aldrich (MO, USA). Recombinant mouse cytokines, interleukin-1beta (IL-1*β*) (401-ML-005), tumor necrosis factor-alpha (TNF*α*) (410-MT-025), and interferon-gamma (INF-*γ*) (485-MI-100), were purchased from R&D Systems (MA, USA). Dihydrorhodamine 123 (DHR) (D23806), BODIPY 581/591 C11, and MitoTracker Red-FM were obtained from Thermo Fisher Scientific (CA, USA). Necrostatin-1 (Nec-1) (sc-200142) was purchased from Santa Cruz Biotechnology (TX, USA). Caspase inhibitor Z-VAD-FMK (Z-VAD) (G7231) was obtained from Promega (MAD, USA). The following antibodies were obtained from Abcam (UK): anti-GPX4 (ab125066), anti-cysteine/glutamate exchanger (xCT) (ab175186), anti-*β*-actin (ab8225), anti-4 hydroxynonenal antibody (4-HNE) (ab46545), Alexa Fluor secondary anti-rabbit antibody (ab150079), and horseradish peroxidase- (HRP-) conjugated secondary anti-rabbit IgG (ab97051). Mouse and rabbit specific HRP/3,3′-diaminobenzidine, DAB (ABC) detection IHC kit (ab64264) was also purchased from Abcam (UK). Anti-superoxide dismutase 1 (SOD1) (sc8637) was purchased from Santa Cruz Biotechnology (TX, USA); anti-phospho NFE2-related factor 2 (pNrf2) (p15-67520) and streptavidin-FITC (SA10002) from Invitrogen, Thermo Fisher Scientific (CA, USA). Anti-insulin antibody (#9016) was purchased from Cell Signaling Technology (MA, USA). Hoechst 33342 dye (910-3015) was purchased from ChemoMetec (Denmark). Rabbit anti-goat IgG-biotin was purchased from Vector Laboratories (BA-5000-1.5) (PA, USA). Mounting media for microscopy slides were Bio Mount Aqua (BMA-100) (BioGnost, Croatia), Fluoromount-G™ Mounting Medium with 6-diamino-2-phenylindole (DAPI) (00-4959-52) (Thermo Fisher Scientific, CA, USA), and Dibutyl Phthalate Polystyrene Xylene (DPX) (06522) (Sigma-Aldrich, MO, USA). Unless otherwise specified, all chemicals and materials used for cell work were purchased from Sigma-Aldrich (MO, USA).

### 2.2. Cell Culture and Treatment

Pancreatic islet tumor Rin-5F (ATCC-CRL-2058) cells were cultivated in RPMI 1640 medium supplemented with FBS (10%), L-glutamine (2 mM), penicillin (100 U/mL), and streptomycin (100 *μ*g/mL), at 37°C, humidity 95%, and 5% CO_2_. Cells were cultured to 80% of confluence, trypsinized, and seeded in 24-well (2 × 10^5^ cells/well) or 6-well plates (8 × 10^5^ cells/well). The cells were treated with (i) RSL-3, an inducer of ferroptosis (3 *μ*M); (ii) high glucose (HG; 25 mM); (iii) STZ (10 mM dissolved in citrate buffer; 0.1 M, pH 4.5); (iv) H_2_O_2_ (75 *μ*M); and (v) cytokines' mixture (IL-1*β*, TNF*α*, and INF-*γ* 20 ng/mL of each) for 12 hours in the presence or absence of 1.5 *μ*M Fer-1, specific inhibitor of ferroptosis. For detection of other types of cell death involvement, instead with Fer-1, cells were treated with specific inhibitors of apoptosis (Z-VAD; 10 *μ*M) and necrosis (Nec-1; 10 *μ*M).

### 2.3. SDS-Polyacrylamide Gel Electrophoresis (PAGE) and Western Blot Analysis

Cultured cells (8 × 10^5^ per well in 6 well plates) were lysed with a buffer containing 62.5 mM Tris–HCl (pH 6.8), 2% SDS, 50 mM DTT, 10% glycerol, and protease inhibitor cocktail. Protein content in the samples was estimated by the method of Lowry et al. [30]. 20 *μ*g of total protein extracts was separated by electrophoresis in 12% SDS-PAGE and transferred onto polyvinylidene fluoride (PVDF) membranes (10600023) (Amersham Hybond P 0.45 PVDF, GE Healthcare Life Sciences, UK). Western blots were performed as described previously [31]. The membranes were blocked for 1 hour at room temperature with 5% nonfat milk in TBS (phosphate-buffered saline, PBS+0.1% Tween 20) and incubated with rabbit polyclonal antibodies against: GPX4 (1 : 1000), SOD1 (1 : 750), xCT (1:1000), and *β*-actin (1 : 2000). The blots were then probed with HRP-conjugated secondary anti-rabbit IgG antibody (1 : 4000). Detection of immunoreactive bands was performed by an enhanced chemiluminescence detection system (sc-2048) (Santa Cruz Biotechnology, TX, USA) using an iBright CL1500 Imaging System (Thermo Fisher Scientific, CA, USA). Quantitative analysis of immunoreactive bands was conducted densitometrically by ImageJ software (National Institutes of Health, MA, USA), as described in more detail previously [27]. The ratio of dots per band for the target protein and *β*-actin (gel loading control) from three independent experiments was averaged, and changes in protein level were expressed as a percentage of an untreated control sample, which was standardized as 100%. Data were then statistically analyzed.

### 2.4. Flow Cytometry

All flow cytometry analyses were performed at least 3 times, with 30,000 cell events analyzed per sample using a CyFlow Space cytometer (Partec, Germany), and data were analyzed using Partec FlowMax® software (Partec, Germany). Experiments were done on cells seeded in a 24-well plate (200,000 cells per well) and treated as described.

#### 2.4.1. Cell Death Assay

After the treatments, cells were washed in PBS, collected by trypsinization, and centrifuged. Cell pellets were resuspended in PBS and immediately stained with 12 *μ*g/mL PI and analyzed on the flow cytometer [32].

#### 2.4.2. Detection of Lipid Peroxides

In order to detect if the experimental treatments used in this study affect lipid peroxidation, a lipid-soluble C11-BODIPY (581/591) fluorescent probe was used, as described earlier [33]. Oxidation of dye molecule results in a shift of fluorescence emission peak from ∽590 nm (red) to ∽510 nm (green). At the end of the treatment period, cells were washed once in PBS, BODIPY 581/591 C11 dye in the RPMI 1640/10% FCS media was added to the final concentration of 2 mM, and cells were incubated for 30 min at 37°C/5% CO_2_ protected from the light. Subsequently, cells were washed 2 times with PBS, detached using trypsin, resuspended in PBS, and analyzed by flow cytometry.

#### 2.4.3. Detection of ROS

ROS was detected by measuring the intensity of green fluorescence emitted by the redox-sensitive dye DHR upon excitation at 488 nm, as described previously [34]. For the measurement of ROS production, cells were seeded in a 24-well plate (200,000 cells per well), incubated with 1 *μ*M DHR for 20 min at 37°C, and treated as described above for 12 hours, followed by PBS washing, trypsinization, and flow cytometry analysis.

#### 2.4.4. Determination of Mitochondrial Membrane Potential

To determine MMP, the MitoTracker® Red FM probe was used as described earlier [35]. After the treatments, cells were washed in PBS, MitoTracker® Red FM dye in RPMI 1640/10% FCS media was added to the final concentration of 50 nM, and cells were incubated for 15 min at 37°C/5% CO_2_, protected from the light. Subsequently, cells were washed 2 times with PBS, detached using trypsin resuspended in PBS, and analyzed by flow cytometry.

### 2.5. Microscopic Analysis

#### 2.5.1. Phase-Contrast Microscopy

For the observation of cell viability, morphology, and confluence, phase-contrast microscope was used (Zoe, BioRad, CA, USA).

#### 2.5.2. Sudan III Detection of Neutral Lipids and Lipofuscin

Rin-5F cells were seeded on sterile coverslips in a 24-well plate and treated as already described. At the end of the treatment period, cells were washed once in PBS, fixed in 4% paraformaldehyde (PFA), and rinsed three times in PBS and two times in distilled water. After the quick rinsing in 50% ethanol, cells were stained with filtered Sudan III working solution (prepared from 2 parts of 0.5% Sudan III in 99% isopropanol and one part of distilled water) for 15 min. After the washing in 50% ethanol and distilled water, cells were counterstained with Mayer's hematoxylin for 3 min, rinsed with distilled water, and mounted on microscope slides after the addition of BioMount aqueous mounting medium. After drying, cells were analyzed on a DMLB light microscope (Leica Microsystems, Germany).

#### 2.5.3. Detection and Localization of Lipid Peroxidation Products

For this experiment, 80000 Rin-5F cells were seeded on eight-well chamber slides (Thermo Fisher Scientific, CA, USA) and treated as previously described. At the end of the treatment period, cells were washed by PBS and incubated with C11-BODIPY for 30 min at 37°C/5% CO_2_ protected from the light. After washing, cells were mounted with Fluoromount and analyzed on a confocal SP5 Leica microscope (Leica Microsystems, Germany). The oxidized form of this fluorescent probe shifts its emission maximum from 590 nm (red) to 510 nm (green). Following excitation/emission detection range settings were applied: 488 nm/500-553 nm and 543 nm/580-620 nm. After adjusting detector gain and offset, all slides were analyzed without additional settings change. In order to quantify the signal intensity of oxidized C11-BODIPY form, an intensity tool of Leica LAS AF software was used. Regions of interest (ROIs) were drawn on acquired micrographs to encompass the cytoplasm of approximately 30 cells per slide. Intensity values were presented as means ± SEM for every group.

#### 2.5.4. Prussian Blue Detection of Intracellular Iron

The intracellular presence of iron was evaluated by Prussian blue (PB) staining. Cells were seeded on sterile coverslips in a 24-well plate, treated, and fixed in 4% PFA as already described. Prussian blue staining was performed by incubating fixed cells in a mixture of equal parts of 20% aqueous solution of hydrochloric acid and 10% aqueous solution of potassium ferrocyanide for 30 min. Afterward, cells were counterstained with nuclear fast red, mounted in DPX on microscope slides, and examined with a Leica DMLB microscope (Leica Microsystems, Germany). The cells exhibiting blue intracellular particles were considered PB-positive, and the percentage of positive cells was determined within the ten randomly selected fields of view per sample.

#### 2.5.5. Immunocytochemical Detection of pNrf2

For immunocytochemical detection of pNrf2, 80,000 Rin-5F cells per well were seeded on eight-well chamber slides (Thermo Fisher Scientific, CA, USA) and treated as previously described. At the end of the treatment, cells were washed once in PBS and fixed in 4% PFA. After washing, cells were permeabilized for 30 min with 0.5% TritonX-100 in PBS. To decrease the nonspecific binding of antibodies, cells were incubated in 5% BSA solution in PBS for 1 hour. Incubation with primary rabbit anti-pNrf2 antibody (diluted 1 : 200 in 1% BSA) was performed overnight at 4°C. After thorough PBS washing, cells were incubated with Alexa Fluor 647 secondary goat-anti-rabbit antibody (diluted 1 : 200 in 1% BSA) for 30 min at room temperature and subsequently washed in PBS. After washing, cells were mounted with Fluoromount-G™ mounting medium with DAPI and analyzed on DM4B fluorescent microscope (Leica Microsystems, Germany). In order to quantify the signal intensity of nuclear pNrf2, an intensity tool of Leica LAS AF software (Leica Microsystems, Germany) was used. ROIs were drawn on acquired micrographs to encompass nuclei of approximately 70-80 cells per treatment. Intensity values were presented as mean ± SEM for every group.

### 2.6. Animals

Male C57BL/6 mice were housed with unlimited access to standard chow and tap water at the animal facility at the Institute for Biological Research “Sinisa Stankovic.” All experimental procedures were approved by the Ethic Committee at the Institute for Biological Research “Sinisa Stankovic” (App. No 01-11/18-01-2476) in accordance with the Directive 2010/63/EU. The 8-10-week-old male C57BL/6 mice were divided into two groups, STZ-treated and STZ + Fer-1-treated. STZ, 40 mg/kg bw, was dissolved in a cold 0.1 M citrate buffer (pH 6) just prior to administration and given intraperitoneally for 5 consecutive days. Fer-1, 1 mg/kg bw, was dissolved in dimethyl sulfoxide (DMSO) first and then diluted in PBS and administered intraperitoneally, starting from the first dose of STZ for 21 days in total. In order to avoid possible interference, the injections of STZ and Fer-1 were given 3 hours apart. The group treated with STZ also received the Fer-1 diluent in equal volume. Both groups consisted of eight animals. After 24 hours of the last STZ and Fer-1 doses (day 22 of the experiment), animals were euthanized; pancreata were collected, embedded in paraffin blocks, and cut into 5 *μ*m thin sections for microscopic analyses.

### 2.7. Microscopic Analysis of Pancreatic Tissue

#### 2.7.1. Histological Stainings and Analyses

For routine histological analysis, pancreata slides were stained routinely with hematoxylin & eosin (HE) stains. Also, AZAN trichrome staining was performed in order to differentiate tissue components according to the routine protocol [36]. Langerhans islet surface was measured in Fiji, an open-source distribution of ImageJ software for biological image analysis (NIH, USA). At least 30 islets per pancreas from three mice per group were analyzed, and the results are presented as mean values ± SEM for every group.

The degree of islet destruction was scored by examining at least 30 islets per pancreas from at least three mice per group and graded in a blinded fashion as follows: healthy islets, with no destruction; peri-insulitis, with mononuclear cells infiltrating the borders of islets; insulitis, with heavy intraislet infiltrate [34]. Results are expressed as a percentage of graded islets out of the total number of islets.

#### 2.7.2. Immunofluorescence Detection of Insulin

To determine the degree of islet cell destruction, pancreata sections were stained with FITC-conjugated rabbit anti-mouse insulin antibody (1 : 400). The nuclei were counterstained with Hoechst 33342 dye (1 *μ*g/mL). The negative control was stained with rabbit anti-goat IgG-biotin coupled with streptavidin-FITC and Hoechst. The slides were examined by fluorescent microscopy (Zeiss Imager Z1, AXIO, Carl Zeiss Meditec AG, Germany). The presence of insulin in the pancreatic islets was determined with Fiji software (NIH, USA) [34]. The acquired images were converted to grayscale, and fluorescence intensity was quantified by measuring the mean gray value, which represents the sum of gray values of all pixels in the selection divided by the number of pixels. Intensity values were presented as means ± SEM for every group.

#### 2.7.3. Immunohistochemical Detection of 4-Hydroxynonenal

For 4-HNE assessment in pancreatic tissue, anti-rabbit 4-HNE antibody (1 : 500) was assessed by the Mouse and Rabbit Specific HRP/DAB (ABC) Detection IHC kit. Primary antibody was paired with a biotinylated secondary antibody (1 : 20), then incubated with HRP-labeled streptavidin (1 : 20), and finally, stained with DAB chromogen solution (all obtained from the ABC kit), while counterstaining was carried out with hematoxylin. For the negative control, the sections were labeled with secondary antibodies only. The sections were examined with a Leica DMLB microscope.

### 2.8. Statistical Analysis

Statistical analysis was performed in GraphPad Prism software (GraphPad Software, CA, USA). To test data for normality, the Kolmogorov–Smirnov test was used. In cases of parametric distribution for *in vitro* experiments, one-way analysis of variance (one-way ANOVA) was performed, or Kruskal-Wallis for nonparametric distribution. If the *F* test showed an overall difference, Bonferonni or Dunn's multiple comparison test was used to evaluate the significance of the differences, respectively. For the *in vivo* experiment, Student's *t*-test was used to evaluate the significance of the differences. Statistical significance was set at *p* < 0.05.

## 3. Results and Discussion

Ferroptosis has been suggested to contribute to pancreatic *β*-cell loss and dysfunction [8, 25, 26]. However, the data connecting HG, lipids, cytokines, and/or ROS levels with ferroptosis of *β*-cells are still obscure. We have found here that mimicking the diabetic microenvironment by HG, STZ, and H_2_O_2_ induced ferroptosis of *β*-cells since commonly used ferroptosis inhibitor, Fer-1, rescued them from death, along with abolishing the effects of those treatments on ROS, lipid peroxides, and iron content. This is consistent with the effect of the treatment with RSL-3, a well-known inducer of ferroptosis. In contrast to its antiferroptotic effects on HG-, STZ-, and H_2_O_2_-treated cells, Fer-1 failed to prevent cell loss induced by proinflammatory cytokines. This implies that cytokine-induced *β*-cells loss is not evidently mediated by ferroptosis. Our results further showed that Fer-1-induced activation of Nrf2 and following increase in GPX4 expression and MMP protected *β*-cells from ferroptosis. This hypothesis on ferroptosis involvement in a *β*-cells loss in diabetes was confirmed in an *in vivo* diabetic model where Fer-1 protected islets from STZ-induced insult and accumulation of lipid peroxides. Taken together, these results suggest that modulation of ferroptotic cell death might be a promising approach for their preservation in diabetes.

### 3.1. Cytotoxic Effects of HG, H_2_O_2_, and STZ on *β*-Cells Involve Ferroptosis

**Figure 1:**
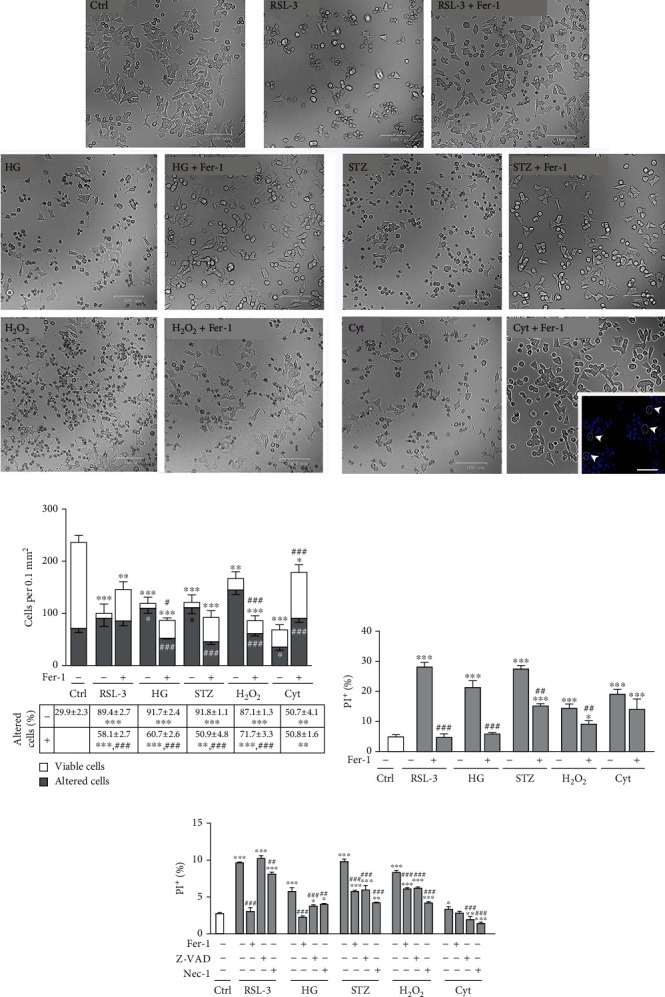
Viability and cell death assessments in Rin-5F cells after 12-hour treatments with RSL-3 (3 *μ*M), high glucose (HG, 25 mM), streptozotocin (STZ, 10 mM), hydrogen peroxide (H_2_O_2_, 75 *μ*M), and proinflammatory cytokines (Cyt, 20 ng/mL) alone or with the addition of ferrostatin-1 (Fer-1, 1.5 *μ*M). (a) Phase contrast microscopy. Insert: DAPI-stained nuclei of Cyt + Fer-1-treated cells with numerous mitotic figures (arrowheads). Orig. magnification: 63x, scale bar (a): 100 *μ*m, insert: 50 *μ*m. (b) Total cell number per 0.1 mm^2^, including the fractions of cells with normal morphology (viable cells) as well cells with altered morphology (rounded, detached, and/or damaged cells). Table: the ratio of altered per total cells' number. (c) Cell death assay: the ratio of propidium iodide (PI^+^) stained cells. (d) Comparison of cell death ratio (PI-stained cells) in the presence of inhibitors of ferroptosis (Fer-1), apoptosis (Z-VAD), and of necrosis (Nec-1). All graph and table values are presented as means ± SEM. Statistical significance: ^∗^in comparison to control: ^∗^*p* < 0.05, ^∗∗^*p* < 0.01, ^∗∗∗^*p* < 0.001; ^#^in comparison to the same treatment without adequate cell death inhibitor (Fer-1/Z-VAD/Nec-1): ^#^*p* < 0.05, ^##^*p* < 0.01, ^###^*p* < 0.001. Significance values above bars: for a total number of cells; significance values on bars: for a number of altered cells. All experiments have been performed in triplicate, and the representative graphs are shown.

Treatments of Rin-5F pancreatic cells with HG, H_2_O_2_, and STZ increased cell death of Rin-5F pancreatic cells (Figure 1). Microscopic analysis (Figures 1(a) and 1(b)) demonstrated a significant decrease of total cell number after HG, STZ (*p* < 0.001), and H_2_O_2_ (*p* < 0.01) treatments in comparison with control (untreated) cells. Majority of cells (over 80%) (*p* < 0.001) after 12-hour treatments changed their shape, from typical multipolar to spherical and detached. Also, large quantities of dead cells and cell fragments are visible in these cultures. Cell death assay confirmed these results, demonstrating the increased number of dead (PI^+^) cells (*p* < 0.001) (Figure 1(c)). When treated with Fer-1, the mentioned effects of HG, H_2_O_2_, and STZ were diminished since the percentage of morphologically altered (HG and STZ, *p* < 0.001; H_2_O_2_, *p* < 0.01), and PI^+^ cells (HG, *p* < 0.001; H_2_O_2_ and STZ, *p* < 0.01) decreased in all cases when compared to the treatments without Fer-1. The alterations induced by HG, H_2_O_2_, and STZ are very similar to those induced by ferroptosis inducer, RSL-3, since it also induced a decrease in cell number (*p* < 0.001) and increase in the percentage of cells with altered morphology (*p* < 0.01) and in cell death (*p* < 0.001), compared to untreated cells (Figure 1). Taken together, these results suggest that the cytotoxic effect of HG, H_2_O_2_, and STZ could be ascribed to ferroptosis of *β*-cells. So far, Fer-1 has been recognized as a powerful tool for verification of ferroptosis [15], and its benefits have been described in the diabetes-related kidney [22, 25, 37], endothelial dysfunction [38], and osteoporosis [24].

Our results further suggest that the contribution of ferroptosis to cytotoxic effects on insulin-producing Rin-5F cells differs between the applied treatments. While Fer-1 fully rescued cells from HG-induced death, by returning PI^+^ cell ratio to control level (*p* < 0.001), similarly as in the case of RSL-3 (*p* < 0.001), this was not the case with H_2_O_2_- and STZ-induced cell death (Figures 1(c) and 1(d)). Namely, although Fer-1 addition decreased PI^+^ cells ratio after STZ and H_2_O_2_ treatments (*p* < 0.01), it did not reach the control level. This indicates that ferroptosis is the predominant cell death of Rin-5F cells treated with HG, but in the case of STZ and H_2_O_2_ treatment, other types of cell deaths have comparable contributions. Cell protective effects of ferroptosis, apoptosis, and necrosis inhibitors Fer-1, Z-VAD and Nec-1, respectively, are similar in STZ- and H_2_O_2_-treated cells (Figure 1(d)). Cytotoxic effects of HG, H_2_O_2_, and STZ have been commonly ascribed to apoptosis or necrosis [14, 39]. However, ferroptotic mode of their cytotoxic actions has also been described—for H_2_O_2_ in rat glioma [40] and cancer cells [41] and for HG in *β*-cells [25, 26]. Our results suggest for the first time ferroptosis as one of the mechanisms of H_2_O_2_- and STZ-induced *β*-cell death.

### 3.2. Lipid Peroxidation/ROS, Iron, and Lipofuscin Accumulation in *β*-Cells Stimulated by Diabetic Environment Correlates with Ferroptosis

**Figure 2:**
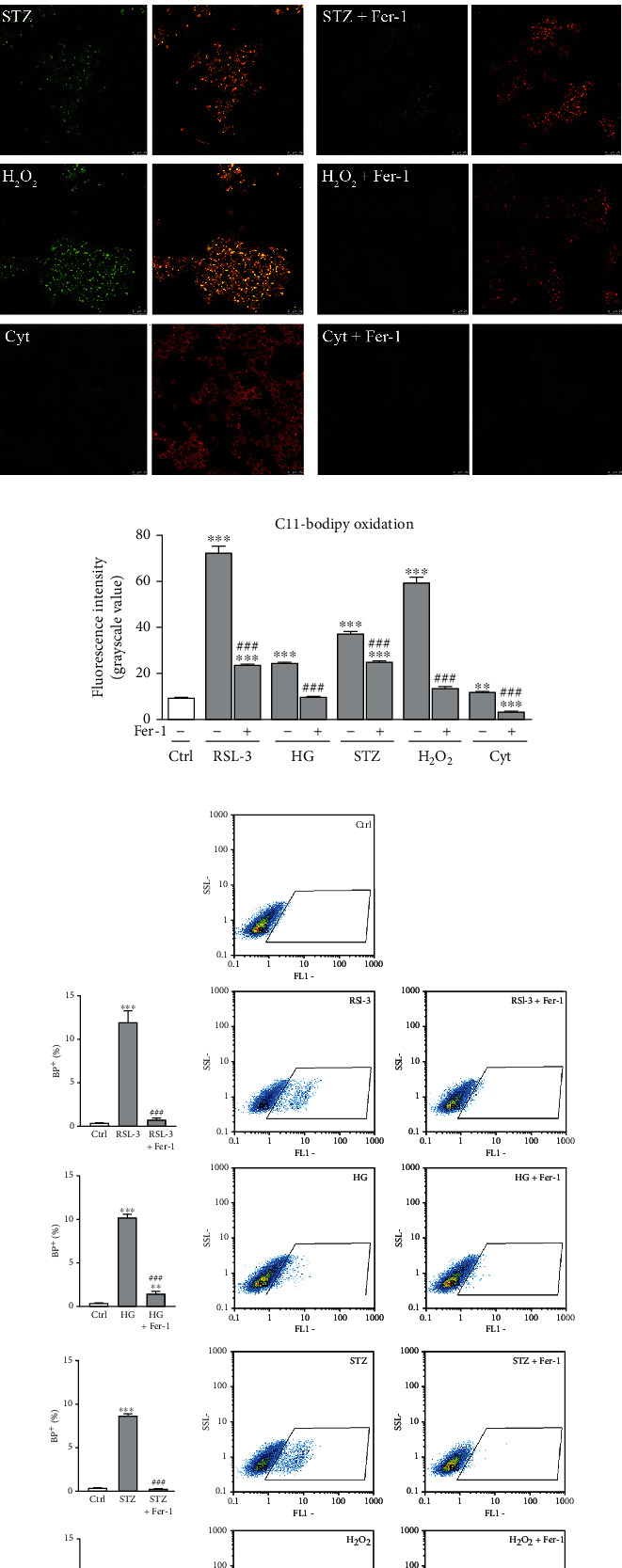
The levels of lipid peroxidation products in Rin-5F cells after 12-hour of RSL-3 (3 *μ*M), high glucose (HG, 25 mM), streptozotocin (STZ, 10 mM), hydrogen peroxide (H_2_O_2_, 75 *μ*M), and proinflammatory cytokines (Cyt, 20 ng/mL) treatments alone or with the addition of ferrostatin-1 (Fer-1, 1.5 *μ*M). (a) Representative images of C11-BODIPY staining obtained by confocal microscopy. Green signal (left image of every pair) represents an oxidized form of C11-BODIPY; superimposed signals of both oxidized (green) and nonoxidated form (red) of the dye are shown as right images of every pair. Orig. magnification: 63x, scale bar: 25 *μ*m. (b) Quantification of emission signal intensity of oxidized C11-BODIPY in Rin-5F cells at confocal microscopy level. (c) Flow cytometric analysis of C11-BODIPY oxidation presented as dot plots and as graphs. All experiments have been performed in triplicate and the representative graphs are shown. All graphs' values are presented as means ± SEM. Statistical significance: ^∗^in comparison to control: ^∗^*p* < 0.05, ^∗∗^*p* < 0.01, ^∗∗∗^*p* < 0.001; ^#^in comparison to the same treatment without Fer-1: ^#^*p* < 0.05, ^##^*p* < 0.01, ^###^*p* < 0.001.

To confirm that HG, H_2_O_2_, and STZ induced ferroptosis of *β*-cells *in vitro*, we examined the effects of the applied treatments on ferroptosis-related parameters. We found that increased death of HG-, H_2_O_2_-, and STZ-treated Rin-5F cells coincided with an increase in the level of lipid peroxides, as detected by the C11-BODIPY probe at confocal microscopy (Figures 2(a) and 2(b)) and flow cytometry (Figure 2(c)) level.

**Figure 3:**
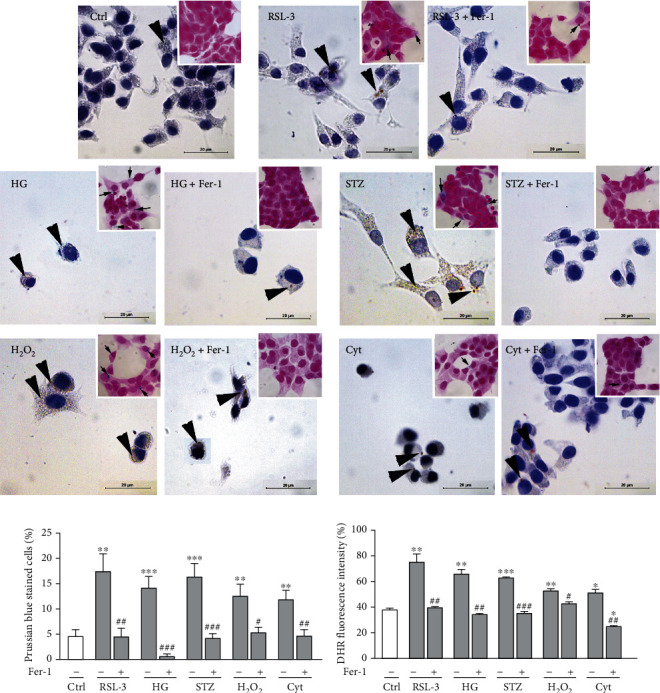
Accumulation of lipofuscin, iron, and ROS in *β*-cells under prodiabetic conditions *in vitro* (Rin-5F cells after 12 hours of RSL-3 (3 *μ*M), high glucose (HG, 25 mM), streptozotocin (STZ, 10 mM), hydrogen peroxide (H_2_O_2_, 75 *μ*M), and proinflammatory cytokines (Cyt, 20 ng/mL) treatments alone or with the addition of ferrostatin-1 (Fer-1, 1.5 *μ*M)). (a) Sudan III staining of neutral lipids and lipofuscin (arrowheads). Inserts: Prussian blue demonstration of ferrous ions accumulation in treated cells (arrows). Scale bars: 20 *μ*m. (b) Quantification of iron-positive cells from Prussian blue-stained samples (inserts in (a)). (c) ROS formation, measured by dihydrorhodamine 123 (DHR) staining. DHR staining has been performed in triplicate, and the representative graph is shown. All graphs' values are presented as means ± SEM. Statistical significance: ^∗^in comparison to control: ^∗^*p* < 0.05, ^∗∗^*p* < 0.01, ^∗∗∗^*p* < 0.001; ^#^in comparison to the same treatment without Fer-1: ^#^*p* < 0.05, ^##^*p* < 0.01, ^###^*p* < 0.001.

As demonstrated in Figures 2(b) and 2(c), a statistically significant increase of C11-BODIPY oxidation was detected in all treated groups when compared to the control (*p* < 0.001). It seems likely that the accumulation of lipid peroxides under these diabetogenic conditions determines cell death and directs it to ferroptosis since the increase in C11-BODIPY oxidation was noticed in the RSL-3-treated group. Also, this is evidenced by the protective effect of Fer-1 which completely prevented the accumulation of lipid peroxides induced by HG, H_2_O_2_, and STZ (*p* < 0.001). The central event in ferroptosis is the uncontrolled accumulation of lipid peroxides due to their increased formation or impaired removal. The main initiators of lipid peroxidation are hydroxyl radical (^**·**^OH) and hydroperoxyl radical (^**·**^OOH) that are formed in the reaction of ferrous iron (Fe^2+^) with H_2_O_2_ known as the Fenton reaction [42], hence the name “ferroptosis.” The diabetic state is characterized by abnormal iron status as has been shown in numerous animal and clinical studies [43–46]. The iron deposition has been observed in various diabetic tissues, including the pancreas, particularly pancreatic islets [44], and correlated with the diabetes-induced pathological changes, including cell death [47]. Since iron represents the key catalyst of Fenton reaction and consequent lipid peroxidation, it is not surprising that diabetes-related iron deposition may lead to ferroptosis. The results of the present study revealed the increase in iron and ROS levels in HG- (*p* < 0.001 and *p* < 0.01, respectively), H_2_O_2_- (*p* < 0.01), and STZ- (*p* < 0.001) treated cells compared to untreated ones (Figures 3(a) (inset), 3(b), and 3(c)). A similar increase was noticed in RSL-3-treated cells. Such effects of HG, H_2_O_2_, and STZ treatments were diminished by Fer-1 co-treatment, seen through the decrease in iron accumulation (HG and STZ, *p* < 0.001; H_2_O_2_, *p* < 0.05) and ROS level (HG, *p* < 0.01; H_2_O_2_, *p* < 0.05; STZ, *p* < 0.001) when cotreated with Fer-1. Increased lipid peroxidation, ROS production, and iron accumulation under the diabetic conditions *in vitro* which are inhibited by Fer-1 confirmed our assumption that ferroptosis is involved in the *β* cell loss.

Along with the increase of lipid peroxides, the cells treated with HG, H_2_O_2_, and STZ have shown the accumulation of neutral lipids and lipofuscin, as demonstrated by Sudan III staining (Figure 3(a)).

Among sparsely distributed lipid droplets, a myriad of small granules corresponding to lipofuscin granules is visible in the cytoplasm of these cells, especially when treated with H_2_O_2_ and STZ. Accumulation of neutral lipids and lipofuscin in these groups is similar to those noticed in RSL-3-treated cells. Lipofuscin is an insoluble aggregate composed of peroxidized lipids, crosslinked proteins' residues, and metals in lysosomes. It is formed due to iron-catalyzed oxidative processes, and its accumulation increases with aging in postmitotic cells of several tissues, such as neurons, brown adipocytes, heart, and skeletal muscle cells [48–51], as well as in various diseases [50, 52]. Lipofuscin is not an inert byproduct of cells, but it rather actively alters cellular metabolism at different levels [53]. It serves as a reservoir of metal ions, including iron, which releases in its reactive form [54] and thus promotes ROS generation and consequently cell death [53]. Its accumulation in *β*-cells under diabetogenic conditions mimicked by HG, H_2_O_2_, and STZ is in line with increased lipid peroxidation, ROS, and iron accumulation and is related to herein demonstrated ferroptotic death of these cells. Namely, Fer-1-induced rescuing of *β*-cells from cell death coincides with the decrease in lipofuscin accumulation. So far, the pathogenic role of ferroptosis in cell death was demonstrated. Also, lipofuscin role in ferroptosis has been recognized in neurons and cardiomyocites [55, 56]. A previous study demonstrated the aging-related accumulation of lipofuscin in human and nonhuman primate *β*-cells [57]. Our study connects lipofuscin accumulation with *β*-cell ferroptotic cell death under diabetic conditions for the first time.

### 3.3. Disturbed Nrf2/GPX4 Axis and Mitochondrial Functional State Correlate with Ferroptosis of *β*-Cells under Diabetic Conditions

**Figure 4:**
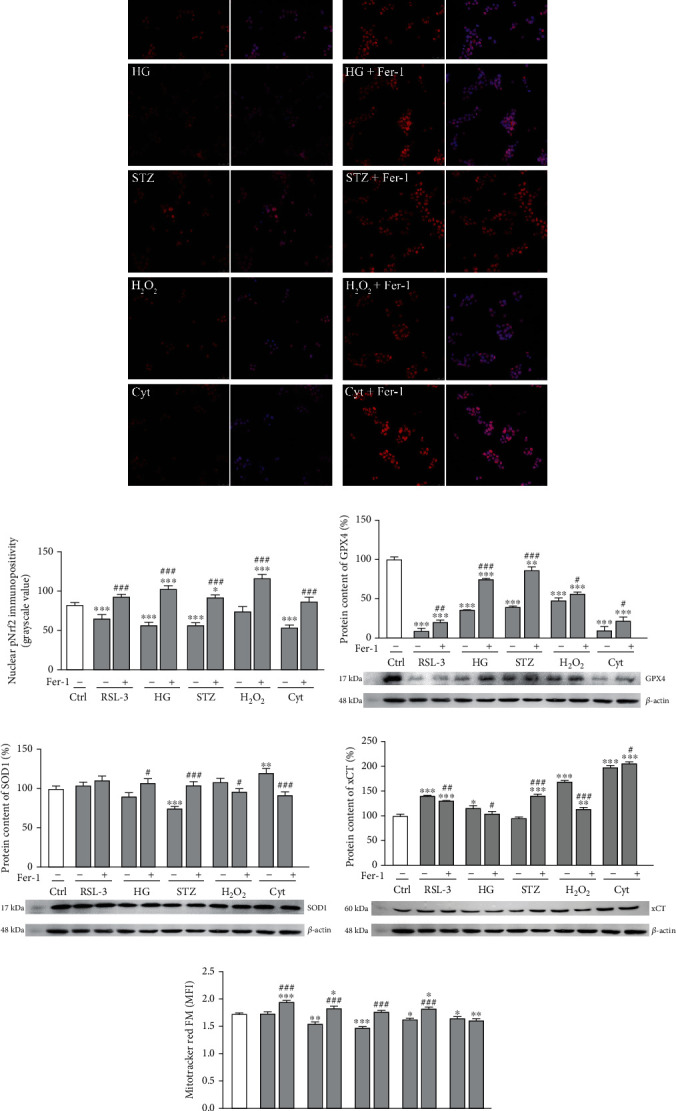
Changes in ferroptosis-related parameters in Rin-5F cells after 12 hours of RSL-3 (3 *μ*M), high glucose (HG, 25 mM), streptozotocin (STZ, 10 mM), hydrogen peroxide (H_2_O_2_, 75 *μ*M), and proinflammatory cytokines (Cyt, 20 ng/mL) treatments alone or with the addition of ferrostatin-1 (Fer-1, 1.5 *μ*M). (a) Microscopic detection of pNrf2 immunopositivity in nuclei. Red signal (left image of every pair) represents pNrf2; superimposed signals of both pNrf2 (red) and DAPI-stained nuclei (blue) are presented at right images of every pair. Orig. magnification: 63x, scale bar: 25 *μ*m. (b) Quantification of pNrf2 nuclear immunopositivity. Changes in protein abundance of (c) GPX4, (d) SOD1, and (e) xCT analyzed by Western blot analysis. *β*-Actin served as a protein-loading control; blots are representatives of three independent experiments. (f) Mitochondrial membrane potential has been detected by MitoTracker Red FM staining. MitoTracker Red FM staining has been performed in triplicate, and representative mean fluorescence intensity (MFI) of MMP is shown. All graph values are presented as means ± SEM. Statistical significance: ^∗^in comparison to control: ^∗^*p* < 0.05, ^∗∗^*p* < 0.01, ^∗∗∗^*p* < 0.001; ^#^in comparison to the same treatment without Fer-1: ^#^*p* < 0.05, ^##^*p* < 0.01, ^###^*p* < 0.001.

In terms of endogenous cellular mechanisms preventing ferroptosis, Nrf2 is an antioxidant transcription factor that has been recognized to be important for the onset and outcomes of ferroptosis [58]. It regulates the level of many of the ferroptosis-related molecules including those involved in the metabolism of GSH (xCT, GPX4, and glutathione reductase), iron (ferritin, transferrin, heme oxygenases, etc.) [59–62], and lipids [63, 64]. The inactivation, inhibition, and knock-down of Nrf2 enhance ferroptosis in a cell [65]. In accordance with these findings, we have found that herein demonstrated ferroptosis of *β*-cells is accompanied by impaired activation of Nrf2. This is demonstrated by decreased nuclear immunopositivity of its active, phosphorylated form in RSL-3- (*p* < 0.001), HG- (*p* < 0.001), and STZ-treated cells (*p* < 0.001) in comparison to control (Figures 4(a) and 4(b)). When cotreated with Fer-1, the level of pNrf2 nuclear immunopositivity increased, thus returning to (RSL-3) or even surpassing (HG-, H_2_O_2_ and STZ-treated Rin-5F cells) the control level. Activation of Nrf2 has been reported as a promising therapeutic target for various diseases including diabetes [65, 66]. In particular, boosting antioxidant defense by Nrf2 activation in *β*-cells with low antioxidative potential [10, 11] seems to be a promising strategy to preserve their function and mass in the context of diabetes [67–70]. Most of these data suggest that Nrf2 activation rescues *β*-cells from apoptosis [71]. The results of the present study suggest that activation of Nrf2 after Fer-1 treatment protects pancreatic *β*-cells against oxidative stress-induced ferroptosis caused by HG, H_2_O_2_, and STZ. It seems likely that Fer-1 releases *β*-cells from high oxidative pressure induced by those treatments thus setting intracellular redox environments appropriately for Nrf2 activation.

Further, we set out to reveal whether the impaired activity of Nrf2 under the diabetogenic conditions and its activation after Fer-1 cotreatment affects its antioxidant downstream targets, SOD1 and GPX4. Similar to RSL-3, no significant alterations of SOD1 protein expression were noted under the tested diabetogenic treatments (HG, H_2_O_2_), except during STZ treatment, where a significant decrease was shown (*p* < 0.001) (Figure 4(d)). The addition of Fer-1 restores SOD1 protein expression to control level in STZ-treated cells and keeps it at control level in other (HG and H_2_O_2_) diabetogenic treatments. An important Nrf2 downstream target relevant for ferroptosis is a member of the GPX family, membrane-associated GPX4 [62]. Our results demonstrated a decrease in its protein expression in HG-, H_2_O_2_-, and STZ-treated cells (*p* < 0.001) compared to untreated Rin-5F cells (Figure 4(c)). When Fer-1 was added over these oxidative stress-inducing treatments, GPX4 protein level increased toward control level (being significantly higher in comparison to adequate treatment without Fer-1: HG, *p* < 0.001; H_2_O_2_, *p* < 0.05; STZ, *p* < 0.001). Similar effects were noted in the case of RSL-3-treatment, whose ferroptotic capacity is based on GPX4 inhibition. GPX4 has been considered to be the primary enzymatic defense mechanism against ROS-mediated membrane peroxides and consequently against ferroptosis due to its strong association with membranes and its close proximity to phospholipid peroxide substrates [72–74]. This has the support in growing data showing that direct inhibition of GPX4 by RSL3 treatment or GPX4-knockout in mice resulted in severe membrane lipid peroxidation culminating ultimately in ferroptotic cell death [17, 73, 75, 76]. In contrast to the other H_2_O_2_ detoxifying enzymes [11, 12], it has been demonstrated recently that GPX4 is highly expressed in insulin-producing cell lines and primary islet cells and that its knockdown caused severe loss of cell viability [77]. This argues that *β*-cells are particularly sensitive to oxidative degradation of membrane lipids and that therefore GPX4 is crucial for their survival. In accordance with this, our results suggest the significance of activation of the Nrf2/GPX4 axis in the protection of *β*-cells from ferroptosis in oxidative/lipid peroxidative stress induced by HG/H_2_O_2_/STZ. Our hypothesis has support in recently published data by Zhou [26] who showed that protective effects of active component in mulberry leaf extracts over HG-induced ferroptosis in INS-1 cells and pancreatic tissue involve activation of Nrf2/GPX4 pathway. In contrast to GPX4, the protein level of xCT was increased in RSL-3-, H_2_O_2_- (*p* < 0.001), and HG- (*p* < 0.01) treated cells compared to untreated cells (Figure 4(e)). The addition of Fer-1 over the above-mentioned treatments decreased protein expression of xCT toward the control level. The observed response of xCT can be explained as a compensatory mechanism activated to import cysteine to maintain GSH homeostasis in such conditions where GPX4 is downregulated. These findings are in accordance with the data of Li et al. [25] who found a similar response of those ferroptosis-related parameters in pancreatic tissue of diabetic animals.

In addition, Nrf2 is critical for mitochondrial functioning, by regulating mitochondrial biogenesis, ATP production, and structural and metabolic remodeling of this organelle [78, 79]. Considering this and the decisive roles of mitochondria for *β*-cell impairment in the progress of diabetes [80], we examined further how mitochondrial membrane potential in cells is changed by applied treatments and whether these changes correlate with localization/activation of Nrf2. Results of Mitotracker Red FM staining (Figure 4(f)) showed that in comparison to control, HG, STZ, and H_2_O_2_ decrease MMP (*p* < 0.01, *p* < 0.001, and *p* < 0.05, respectively), while Fer-1 restored it (in STZ-treated cells) or even increased it above the control (in HG- and H_2_O_2_-treated cells) (*p* < 0.05). The fact that Fer-1 rescued MMP in Rin-5F pancreatic cells strongly suggests that mitochondrial impairment is an important event underlying the ferroptotic death of *β*-cells in diabetic conditions mimicked by HG, STZ, and H_2_O_2_. This is in agreement with the data of Jang et al. [81] who reported that diminished mitochondrial bioenergetics by ferroptotic stimuli is an important step in ferroptosis-related cell death and also with the data of others showing preservation of MMP by ferroptosis inhibitors [8, 82, 83]. The observed improvement of MMP by Fer-1 coupled with activation of Nrf2 reveals the involvement of mitochondria in diabetes-related ferroptosis of pancreatic *β*-cells and suggests the importance of Nrf2 in the regulation of mitochondrial functioning in this type of cell death.

### 3.4. Loss of *β*-Cells Induced by Proinflammatory Cytokines Does Not Involve Ferroptosis

Islet-infiltrating macrophages and T lymphocytes are involved in *β*-cell destruction during the pathogenesis of type-1 diabetes since they release proinflammatory cytokines such are IFN-*γ*, IL-1*β*, and TNF*α* [84]. The mechanism of their detrimental effects in *β*-cells involves increased ROS generation, endoplasmic reticulum stress, and mitochondrial dysfunction. This leads to intrinsic apoptosis, which is considered as the main mechanism of the death of *β*-cells induced by cytokines [85–87]. To reveal if ferroptosis also contributes to cytokine-induced removal of *β*-cells, Rin-5F cells were treated with the mixture of IL-1*β*, TNF*α*, and IFN-*γ*. In contrast to HG, H_2_O_2_, and STZ for which we clearly demonstrated to induce ferroptosis of *β*-cells, our results suggest that ferroptosis is not contributing, at least not evidently, to the cytokines-induced Rin-5F cells loss. Proinflammatory cytokines treatment significantly decreased total Rin-5F cell number in comparison to control (*p* < 0.001) (Figure 1(b)). Along with this, there was an increased level of lipid peroxidation (*p* < 0.01/0.001; Figures 2(b) and 2(c)), iron (*p* < 0.01; Figure 3(b)), and ROS (*p* < 0.05; Figure 3(c)). Also, decreased pNrf2 nuclear immunopositivity (*p* < 0.001; Figures 4(a) and 4(b)), protein expression of GPX4 (*p* < 0.001; Figure 4(c)), and MMP (*p* < 0.05; Figure 4(f)) were demonstrated in cytokine-treated cells when compared to the control. Fer-1 addition abolished these effects by returning these toward control levels (lipid peroxidation *p* < 0.001, ROS *p* < 0.01, iron *p* < 0.01, pNrf2 *p* < 0.001, and GPX4 *p* < 0.05) except for MMP level, since it remains decreased. It is worth mentioning that the lipid peroxidation level induced by cytokines is notably lower than in RSL-3-treated cells. Besides, although a very significant decrease of cell number occurred under the cytokines' treatment, only about 50% of morphologically altered cells and 20% of dead PI^+^ cells were detected (Figures 1(b)–1(d)). These levels are significantly lower when compared to those induced by RSL-3, HG, H_2_O_2_, and STZ. After the addition of Fer-1, the percentage of morphologically altered and dead PI^+^ cells remained unaltered (Figures 1(b)–1(d)). The inability of Fer-1 to improve cell viability and MMP is in accordance with recently published data by Krummel et al. [77] in which was showed that cytokine-induced cell death of *β*-cells did not involve ferroptosis, since it is inhibited by increased nitric oxide (NO) production [88]. However, although Fer-1 treatment failed to prevent cell death of Rin-5F, it significantly improved their proliferation rate, since numerous mitotic figures were detected when cotreated with proinflammatory cytokines and Fer-1 (Figure 1(a)-insert). This significantly contributes to increased cell abundance (seen as increased cell number in Figure 1(b) (*p* < 0.001)). This sheds light on the antiproliferative aspect of proinflammatory cytokine action in *β*-cell loss which is much less explored than their cytotoxic effect [89]. Also, the fact that Fer-1 improved the proliferation capacity of *β*-cell, probably through the radical-trapping and therefore improving cell viability, suggests the possibility of its usage in the prevention and/or the treatment of diabetes. As we demonstrated herein, it could diminish the *β*-cell loss induced by diabetogenic milieu (HG, H_2_O_2_) but could also improve their regeneration by inhibiting the antiproliferative activity of toxic proinflammatory cytokines in pancreatic islets.

### 3.5. Inhibition of Ferroptosis Achieved Beneficial Effects on Pancreatic Islets Challenged by Diabetogenic Stimuli *In Vivo*

**Figure 5:**
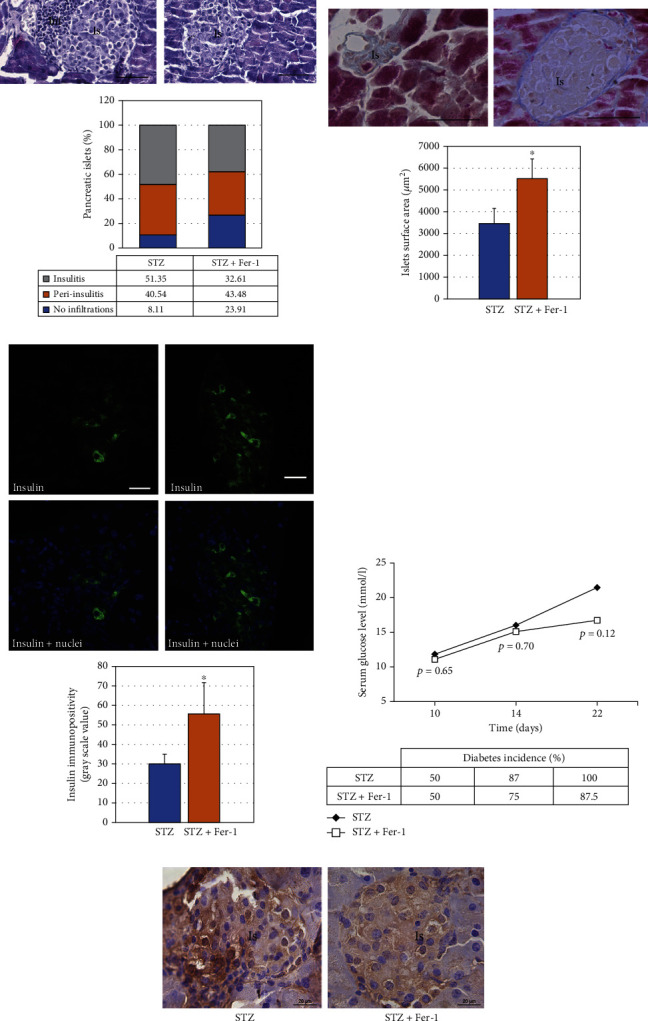
The effects of Fer-1 *in vivo*. (a) Representative HE micrographs of islets and the proportion of pancreatic islets without immune cell infiltrates (inf), with infiltrates surrounding the islets (peri-insulitis) and with infiltrates within the islet (insulitis) in mice treated with STZ and STZ + Fer-1 for 21 days. (b) Representative trichrome-stained micrographs of islets (Is) and average islet surface area in mice treated with STZ and STZ + Fer-1 for 21 days. (c) Insulin immunopositivity of *β*-cells from mice treated with STZ and STZ + Fer-1 for 21 days—representative images of pancreatic islets, stained for insulin visualization (green) and with Hoechst 33342 (nuclei—blue) and insulin immunofluorescence intensity. (d) Serum glucose levels and diabetes incidence in STZ and STZ + Fer-1-treated mice monitored at different time points (10, 14, and 22 days from initial STZ and SZT + Fer-1 dose). (e) Immunohistochemical staining of 4-HNE in the pancreas of mice treated with STZ and STZ + Fer-1 for 21 days. Graphs' values are presented as means ± SEM; statistical significance—comparison of STZ + Fer-1-treated animals with STZ control: ^∗^*p* < 0.05. Orig. magnification and scale bars: (b) and (d) 40x, 50 *μ*m; (c) and (e) 63x; 20 *μ*m.

To further examine Fer-1-mediated protection against diabetogenic stimuli, we used an *in vivo* model of STZ-induced damage to the pancreas. The analysis of mononuclear cell infiltration after the treatments (at day 22) revealed that the islets of Fer-1-treated animals consistently contained fewer mononuclear cells compared with STZ controls (Figure 5(a)), resulting in a higher number of intact islets. Recruitment and activation of mononuclear cells in pancreatic islets induce local production of inflammatory cytokines [90]. They contribute to *β*-cell death *in vivo* as evidenced in cytokine blockade studies in the multiple low-dose STZ model of hyperglycaemia [91, 92]. In addition to decreased mononuclear cell infiltration, statistically larger islets' surface area in Fer-1-treated diabetic animals was demonstrated (Figure 5(b)).

These results are in line with the higher presence of functional insulin^+^*β*-cells in the Fer-1-treated group (Figure 5(c)). As a result, a tendency of decrease of serum glucose level (at day 22 of experiment), accompanied by decreased incidence of hyperglycaemic animals (detected at day 14 and 22 of experiment) were noted in the Fer-1-treated diabetic animals compared to those that were not treated with this compound (Figure 5(d)). Altogether, these data indicate that (21 day) Fer-1 treatment exerted protective effects on the pancreatic islets challenged by diabetogenic stimuli *in vivo* and suggest to examine longer Fer-1 treatment.

Furthermore, the results of 4-HNE immunopositivity speak in favor of the involvement of ferroptosis in *β*-cells demise in diabetic islets. 4-HNE is the second product of lipid peroxidation, and it represents the major readout of increased lipid peroxidation in *β*-cells in diabetic states [93] and of ferroptosis itself [94]. Lower immunopositivity of 4-HNE was observed in the pancreatic islets of Fer-1-treated animals in comparison to STZ-treated diabetic control (Figure 5(e)). These results suggest that Fer-1 decreases the accumulation of lipid peroxides in diabetic islets.

**Figure 6:**
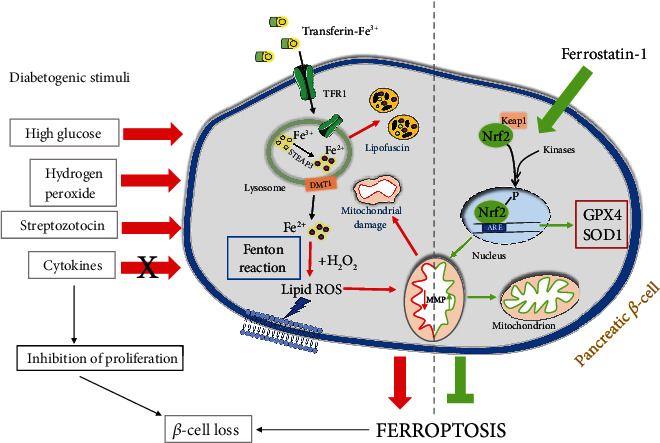
Graphically summarized finding of the current manuscript.

Altogether, these results are in line with the results obtained in our *in vitro* study and confirm positive effects of the inhibition of ferroptosis in *β*-cells' protection and survival under diabetic conditions. The results from the present study are graphically summarized on Figure 6.

## 4. Conclusion

In conclusion, our study demonstrates that *β*-cells undergo ferroptotic cell death under diabetogenic conditions. Precisely, the results suggest that HG and increased ROS production, but not proinflammatory cytokines, are the contributing factors to ferroptosis in the diabetic environment. Also, we suggest that ferroptosis represents the novel mechanism of STZ-induced *β*-cell death. Furthermore, the results shed new light on an antidiabetic strategy based on Nrf2 activation, putting it into the context of protection from ferroptosis. Overall, the findings of this study suggest that targeting ferroptosis in diabetic conditions represents a new scientific avenue that has to be followed in the efforts for the improvement of antidiabetic therapy. Here, presented results from *in vivo* study justify this approach.

## Data Availability

Data are available on request.
